# A scalable cognitive behavioural therapy intervention for perinatal insomnia: a protocol for a hybrid effectiveness-implementation type 1 randomised controlled trial

**DOI:** 10.1186/s13063-025-09308-5

**Published:** 2025-12-30

**Authors:** Meagan E. Crowther, Orly Atzmon, Christie J. Bennett, Margot Davey, Sean P. A. Drummond, Rachel Manber, Ben W. Mol, Duncan Mortimer, Denise A. O’Connor, Daniel L. Rolnik, Jenny Ryan, Joshua F. Wiley, Bei Bei

**Affiliations:** 1 Turner Institute for Brain and Mental Health, School of Psychological Sciences, Faculty of Medicine, Nursing and Health Sciences, Monash University, Melbourne, VIC Australia; 2 Department of Nutrition, Dietetics and Food, School of Clinical Sciences, Monash University, Melbourne, VIC Australia; 3 Melbourne Children’s Sleep Centre, Monash Children’s Hospital, Monash Health, Melbourne, VIC Australia; 4 Department of Psychiatry and Behavioral Sciences, School of Medicine, Stanford University, Stanford, CA USA; 5 Department of Obstetrics and Gynaecology, Monash University, Melbourne, VIC Australia; 6 Centre for Health Economics, Monash University, Melbourne, VIC Australia; 7 School of Public Health and Preventive Medicine, Monash University, Melbourne, VIC Australia; 8 Royal Women’s Hospital, Melbourne, VIC Australia

**Keywords:** Insomnia, Sleep, Pregnancy, Postpartum, Perinatal, Cognitive behavioural therapy, Sleep hygiene

## Abstract

**Background:**

Insomnia symptoms during the perinatal period are prevalent and may contribute to negative mental health and birthing outcomes. Cognitive Behavioural Therapy for Insomnia (CBT-I) is a non-pharmacological therapy efficacious in the treatment of insomnia. Previous studies have shown the effectiveness of digital CBT-I during the perinatal period. However, to date, our understanding of whether this treatment can be effectively implemented in community perinatal care is limited.

**Methods:**

In this two-arm hybrid effectiveness-implementation type 1 randomised controlled trial (RCT), eligible pregnant individuals with self-reported insomnia symptoms (Insomnia Severity Index > 7) will be randomised to either the CBT-I intervention (Healthy Sleep Program) or active control (sleep hygiene education). The primary outcome is maternal insomnia symptom severity at (i) one pregnancy endpoint and (ii) averaged across three times post birth for the postpartum endpoint. An economic evaluation will assess cost-effectiveness. Barriers and enablers to sustained implementation will be explored using the Theoretical Domains Framework and the Practical Robust Implementation and Sustainability Model.

**Discussion:**

This study will offer an understanding of the effectiveness, cost-effectiveness, and sustained implementation potential of a digital sleep health program in perinatal care. These outcomes will provide empirical evidence to inform broader implementation of a scalable sleep program to improve insomnia symptoms in perinatal populations.

**Trial registration:**

Australian New Zealand Clinical Trials Registry ACTRN12622000940774. Registered on 1 July 2022.

**Supplementary Information:**

The online version contains supplementary material available at 10.1186/s13063-025-09308-5.

## Introduction

### Background and rationale {9a}

Sleep disturbance is prevalent during the perinatal period, with about 70% of individuals experiencing sleep disturbance during pregnancy [1], and a similar proportion (66%) of birthing parents continuing to experience sleep complaints well into the first year postpartum [1]. These sleep problems may be caused by factors that disrupt sleep (e.g. physical discomfort, hormonal changes, night-time infant care), psychosocial stress [2, 3], and symptoms of insomnia. Insomnia symptoms are characterised by difficulty initiating and/or maintaining sleep or early morning awakenings [4], and unlike sleep disruption, insomnia symptoms persist despite adequate and undisrupted sleep opportunity (e.g. when not woken for caregiving) [5]. While sleep disturbance and dissatisfaction with sleep are often considered a normal part of the perinatal period, about one in three individuals have sleep complaints that may require clinical attention [5].

Insomnia and sleep disruption in perinatal populations have been associated with mood disturbances [6], impaired daytime functioning [7], symptoms of depression and anxiety, increased risk for postpartum depression [8], and heightened probability of motor vehicle accidents [9]. Additionally, perinatal insomnia and sleep disturbance are associated with poorer pregnancy outcomes, including gestational diabetes [10], unplanned caesarean birth [11], and increased risk of preterm birth [12, 13].

Considerable evidence from multiple systematic reviews and meta-analyses [14, 15] has led to the consensus that Cognitive Behavioural Therapy for Insomnia (CBT-I) is a first-line intervention for people experiencing insomnia and sleep complaints [16]. In addition to its demonstrated effectiveness in treating insomnia symptoms, CBT-I has additional benefits, including reducing depression and anxiety symptoms [17]. Previous randomised controlled trials (RCTs) have demonstrated the effectiveness of CBT-I for use in perinatal insomnia, with meta-analysis indicating a significant reduction in insomnia severity with high-quality evidence [18]. When initiated during pregnancy, CBT-I has also been demonstrated to increase the likelihood of insomnia remission [19, 20], to significantly reduce insomnia symptoms [21, 22] and to prevent postpartum insomnia [22]. Additionally, studies also demonstrate that CBT-I delivered digitally is beneficial for insomnia symptoms during the perinatal period [21, 22]. At present, CBT-I is not part of standard perinatal care in Australia, and there is a need for further exploration of the effectiveness, cost-effectiveness, and implementation potential of this intervention [23] in the broader community and healthcare settings. Therefore, the present study will examine the effectiveness, cost-effectiveness, and implementation factors of a CBT-I-based digital intervention in the perinatal period.

Furthermore, there is limited understanding of what factors contribute to the emergence of parental sleep problems during early parenthood and little consideration of perinatal sleep disturbance in fathers and partners [24]. Thus, the present study also has two auxiliary studies to examine changes in sleep and wellbeing in pregnant individuals who do not report high insomnia symptoms and sleep and wellbeing changes in fathers and non-birthing parents (supplementary material).

### Explanation for the choice of comparator {9b}

A CBT-I based Healthy Sleep Program has been chosen as the intervention condition given its effectiveness in reducing insomnia symptoms in previous trials [21, 22]. An active control of sleep hygiene education has been chosen for its well established face validity of a sleep intervention and to control for non-specific effects (e.g., contact with researchers, participating in a sleep trial). All participants will continue to receive perinatal care as usual.

### Objectives {10}

#### Aim 1

To evaluate the clinical effectiveness of a digital CBT-I based Healthy Sleep Program against an active control condition. The primary outcome for effectiveness is symptoms of insomnia. The secondary outcome is sleep-related impairment.

#### Aim 2

To conduct a health economic evaluation comparing Healthy Sleep Program to sleep hygiene education, examining (1) whether cost savings from reduced health service utilisation offset the direct cost of the intervention and (2) if not cost saving whether the hypothesised improvements in sleep and daytime functioning are worth the additional cost of the intervention.

#### Aim 3

To describe the acceptability and appropriateness, and to explore the barriers and enablers to sustained implementation of the Healthy Sleep Program in the community using qualitative semi-structured interviews with treating clinicians, participants and their partners, and other key stakeholders.

## Methods: patient and public involvement and trial design

### Patient and public involvement {11}

The study is informed by a Community Reference Committee which involves lived experience advisors (mothers, fathers, and professionals who work with individuals during the perinatal period) (*N* = 5) who provided insight prior to study commencement. The Community Reference Committee completed consumer involvement training, attended at least two meetings, and provided feedback on intervention material, study questionnaires, and recruitment strategies. The Community Reference Committee will also be involved in the development of dissemination materials after the conclusion of the study. The Community Reference Committee members are provided with an AU$50 gift card or reimbursement for each hour involved in the study.

### Trial design {12}

The Sleep Health in Perinatal Care (SHINE) study is a two-arm hybrid effectiveness-implementation type 1 superiority RCT [25], designed to test a clinical intervention while also gathering information about its delivery and implementation potential (Aims 1, 2, and 3). There will be two additional auxiliary observational studies which are described in the supplementary material.

## Methods: participants, interventions, and outcomes

### Trial setting {13}

The trial will be conducted in the community in Australia, with participants recruited from two large Victorian public hospitals (Monash Health and Royal Women’s Hospital) and through community recruitment.

### Eligibility criteria for participants {14a}

**Fig. 1:**
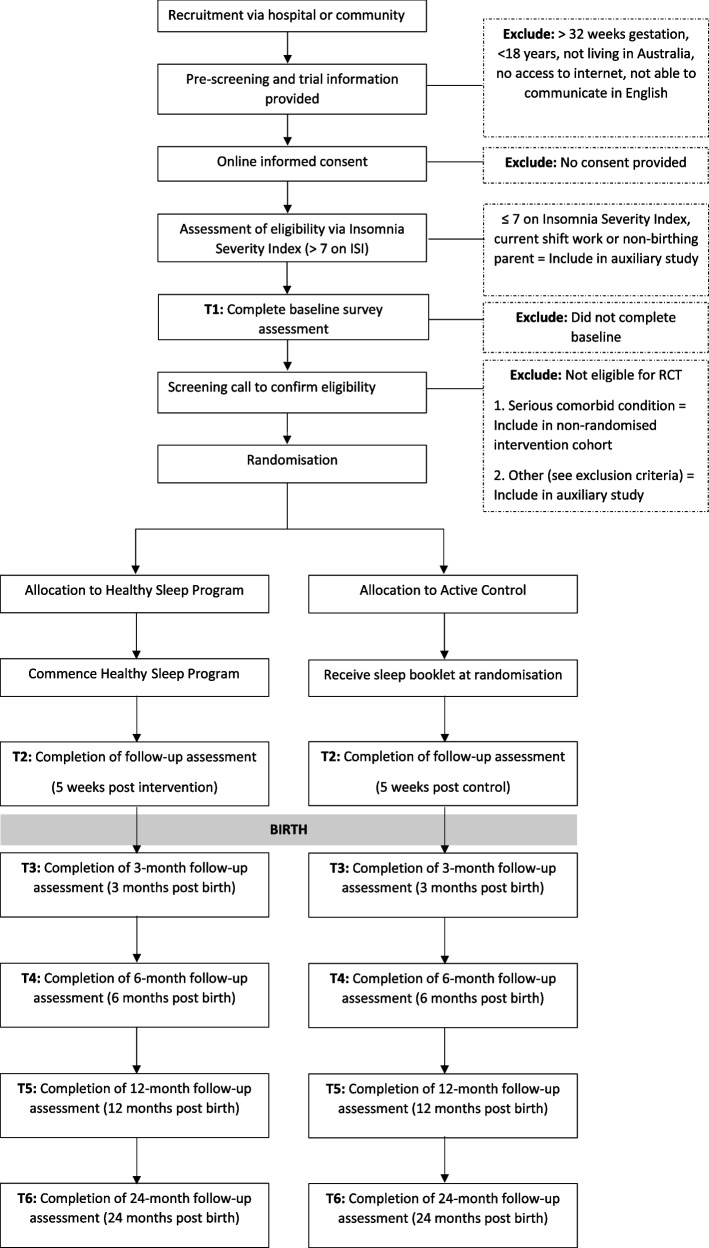
Flowchart of study recruitment, eligibility assessment, randomisation and data collection procedures

The study is a two-arm RCT. Additionally, there is also one intervention-only cohort (described below). In addition to the RCT, there will be two auxiliary studies that are described in the supplementary material. The participant flow is outlined in Fig. 1.

#### RCT cohort

The RCT cohort includes at least 384 pregnant individuals who meet the following inclusion criteria:

1. Expectant mothers and birthing parents aged 18 years or older
2. At least 26 weeks but no more than 32 weeks of gestation at enrolment
3. Able to communicate (read/write/speak) in English
4. Have regular access to e-mail and the Internet
5. Currently live in Australia
6. Score > 7 on Insomnia Severity Index (ISI) [26] at baseline

Participants meeting any of the following exclusion criteria will be excluded from the RCT cohort:

1. Participants with stable use (five or more times per month) of medications or substances (prescription and over the counter) that directly affect sleep and non-stable use will be assessed on a case-to-case basis. Stable use of psychotropic medications for the treatment of non-sleep-related conditions (e.g. antidepressants, anxiolytics) is not exclusionary, and any changes in doses will be documented
2. Participants with unstable medical conditions (e.g. severe diabetes, reflux) that directly and significantly affect sleep will be excluded. Medical conditions that do not directly or significantly impact sleep are not exclusionary
3. Participants with mental health conditions that significantly affect sleep, including severe current posttraumatic stress disorder, current substance abuse/dependence disorders, lifetime bipolar or psychotic disorders, and current suicidal ideation/self-harm behaviours or individuals who pose a risk of harm to others
4. Participants who self-report a current diagnosis of the following sleep disorders, with symptoms significantly affecting sleep:
  - Sleep apnoea: Loud snoring, observed gasping or pauses in breathing, or previously diagnosed with apnoea/hypopnea index > 15/h but not treated or inadequately treated
  - Periodic limb movement disorder with arousal index > 15 per hour
  - Self-reported diagnosis of restless legs syndrome occurring three times/week, with duration of at least 1 month and onset prior to pregnancy. Not exclusionary if restless leg syndrome increased or emerged during pregnancy (as long as pre-pregnancy frequency was no more than once a week)
  - Severe circadian rhythm disorders: Irregular sleep-wake disorder, non-24-h sleep-wake syndrome, advanced sleep-phase syndrome (if habitual bedtime is earlier than 8 pm and habitual wake time is earlier than 4 am, occasional deviation from this schedule is allowed), and delayed sleep-phase syndrome (if habitual bedtime is later than 3 am and habitual wake time is later than 11 am, occasional deviation from this schedule is allowed)
  - Narcolepsy
  - Other previously diagnosed sleep disorders if severe, assessed on a case-to-case basis
5. Participants who are undertaking fixed night shift work (between midnight and 5 am) or rotating work schedules that require night shifts at enrolment

#### Intervention-only cohort

To facilitate the interpretation of findings from Aims 1, 2, and 3 and to examine the feasibility and potential effects of the intervention in vulnerable individuals with *ISI* > 7 who will be excluded from the RCT due to co-existing mental and/or health conditions (see exclusion criteria), we will provide these individuals with the Healthy Sleep Program intervention and conduct the same assessments as for participants in the intervention arm of the RCT.

If participants are excluded from the RCT due to the following exclusion conditions and are currently engaged with a health care provider for their condition/s, they will be included in the “Intervention-only cohort” of the study (i.e. they will not be randomised):

1. Current posttraumatic stress disorder
2. Current substance abuse/dependence disorder
3. Lifetime bipolar or psychotic disorders
4. Medical conditions that seriously and significantly impact sleep
5. Current suicidal ideation/self-harm behaviours or posing a risk of harm to others will be referred to appropriate services and could re-engage with the research project once the risk is managed and mental health support is established

#### Screening

After the baseline survey, a trained researcher will conduct a screening call to further assess eligibility. Participants will be asked to provide further detailed information regarding medication use, mental health conditions, serious health conditions, and suicide ideation. A mental health risk assessment will also be conducted (supplementary material: Mental health protocol). At the conclusion of this screening call, if the participant meets the criteria for the RCT cohort, they will be randomised.

### Eligibility criteria for sites and those delivering interventions {14b}

To be eligible to deliver intervention, the clinician must be a registered or provisional psychologist having completed training developed by the study PI, and receive ongoing supervision for intervention delivery.

### Who will take informed consent {32a}

Participants will be provided with a participant information and consent form (PICF) via Research Electronic Data Capture (REDCap). Study data, including informed consent, will be collected and managed using REDCap electronic data capture tools hosted at Monash University [27, 28].

Participants will provide informed consent to participate in the study through an online consent form. Participants will be informed that questionnaires will collect information about themselves and their infant and will also be asked for consent to collect information from medical records (such as their health during pregnancy and birth and the neonate’s weight and length). Following consent, participants receive a copy of the PICF via email. The PICF indicates that de-identified data may be used in other studies. Additionally, participants can choose to opt in to being contacted about future research.

### Additional consent provisions for collection and use of participant data and biological specimens in ancillary studies {32b}

No biological specimens are collected in the present trial. All participants in the auxiliary studies (supplementary material) will provide informed consent.

## Intervention and comparator

### Intervention and comparator description {15a}

#### Healthy sleep program

The Healthy Sleep Program will use therapist-assisted self-help digital Cognitive Behavioural Therapy for Insomnia (CBT-I) adapted from our previous CBT-I interventions [21, 22]. The Healthy Sleep Program aims to address three types of perinatal sleep complaints: (1) symptoms of insomnia, (2) pregnancy and infant-related sleep disturbance, and (3) sleep-related daytime impairment (e.g. sleepiness and fatigue).

Participants randomised to the Healthy Sleep Program will receive the following content, which is delivered via the following means, combined:

1. A 60-min standardised telephone or telehealth session is delivered by a trained researcher at program entrance to (a) introduce core components (factors contributing to sleep, managing insomnia and sleep deprivation, addressing unhelpful thoughts/beliefs about sleep), (b) discuss personalised strategies using intervention materials (additional components that are relevant to each individual will be added, such as sleep restriction, managing night-time worries, managing physical discomfort), and (c) motivate adherence and sustainable behavioural change. Only key components and those relevant to the individual (i.e. not all components) will be delivered in this session.
2. Up to three optional mini-consultations via phone or telehealth (~15 min each) before childbirth if required
3. One mini-consultation via phone or telehealth (~30 min) when the newborn is 3 months
4. Partner or another person who will also be involved in caring for the newborn is encouraged to attend at least one of the above consultations
5. Multimedia intervention materials (including written text, images, and audio) are delivered digitally via email at enrolment, 5 weeks after enrolment, and 2 weeks, 1.5 months, 3 months, and 6 months postpartum. These electronic materials are timed according to probable sleep challenges at each stage of the perinatal period (e.g. managing insomnia, physical discomfort, and expectation of postpartum sleep at late pregnancy and managing daytime sleepiness during early postpartum). Each electronically delivered module is designed to be succinct and easy to read on a computer, tablet, or phone and will take no more than 10 min to read. Content accessing information such as email opening rate will be captured to assess compliance

Participants who require additional support applying intervention materials can request brief email or telephone clarification from our team. All sessions will be audio recorded for assessing treatment fidelity.

### Materials

The following evidence-based therapeutic components are included in therapy sessions and through multimedia content as follows:

1. General skills to increase resilience to sleep challenges, such as sleep hygiene, sleep education, addressing unhelpful thoughts and beliefs about sleep and relaxation
2. Identifying and managing insomnia symptoms, including stimulus control and time-in-bed restriction (when necessary)
3. Fostering realistic expectations and normalising some sleep loss via early education on sleep patterns of new parents and infants
4. Mindfulness-based strategies targeting physical discomfort, pain, and cognitive arousal
5. Age-appropriate and evidence-based infant sleep/settling skills to reduce awakenings and increase maternal sense of control
6. Prioritising one’s own sleep and rest
7. Smart naps based on circadian principles
8. Managing sleepiness/fatigue
9. Enlisting support
10. Avoiding the supine going-to-sleep position in pregnancy to reduce risk for late stillbirth

Modifications are made to adapt these components to the perinatal periods. For example, sleep education included sleep patterns of new parents and infants, avoiding the supine going-to-sleep position in pregnancy to reduce the risk of late stillbirth [29], and age-appropriate infant sleep/settling skills; relaxation and mindfulness-based strategies targeted physical discomfort, pain, and cognitive arousal; managing sleep loss such as prioritising sleep and rest, naps based on circadian principles, managing sleepiness/fatigue, enlisting support, and discussions with partner and/or family members regarding expectations and responsibilities related to infant sleep and infant care; and, further, an emphasis is placed on differentiating insomnia and sleep deprivation and choosing appropriate strategies.

### Therapists

Therapists will be provisional psychologists and registered psychologists with experience in cognitive behavioural therapy delivery and have undergone training for the Healthy Sleep Program.

### Training and supervision of therapists

All therapists will be trained and supervised by experienced cognitive behavioural therapists. Therapists will be offered fortnightly supervision with the principal investigator.

### Fidelity

All sessions will be audio recorded for assessing treatment fidelity.

#### Active control condition

Sleep hygiene condition

The sleep hygiene condition will account for the non-specific effects of attention and participation in a sleep program (e.g. receiving health information and expectations of benefits). Participants in the control condition will receive an information booklet at program entry containing the same sleep hygiene education as included in the Healthy Sleep Program but not including any other Healthy Sleep Program components. This booklet will be based on nonactive components of a previously tested intervention [21, 22].

### Criteria for discontinuing or modifying allocated interventions {15b}

Participants will discontinue intervention and assessments if their pregnancy has resulted in pregnancy loss or their newborn has died. All data collected from these participants may still be used in data analysis, as outlined in the “Effectiveness for Aim 1” section. Participants will also be free to withdraw from receiving their allocated intervention, in which case we will encourage their ongoing participation in completing relevant follow-up assessments. Regardless of withdrawal status, all data collected from these participants may still be used in data analysis, as outlined in the “Effectiveness for Aim 1” section.

### Strategies to improve adherence to intervention/comparator {15c}

The intervention will be delivered by videoconferencing and/or telephone. Additionally, multimedia content is sent via e-mail. Participants will be contacted at least three times if they have not attended or booked an appointment with the therapist. For the control condition, participants are encouraged to implement the strategies from the booklet to support their sleep. Participants in both groups are encouraged to share these materials with their partner or support person to assist in adherence.

### Concomitant care permitted or prohibited during the trial {15d}

All participants will receive usual perinatal care. Participants in either arm of the study are not prohibited from undertaking any treatment during trial participation.

### Ancillary and posttrial care {34}

All participants will be provided with the contact information of the chief investigator for future contact if such needs arise. Participants in the control condition will receive all Healthy Sleep Program materials (without therapist appointments) following the completion of the trial.

### Outcomes {16}

**Fig. 2:**
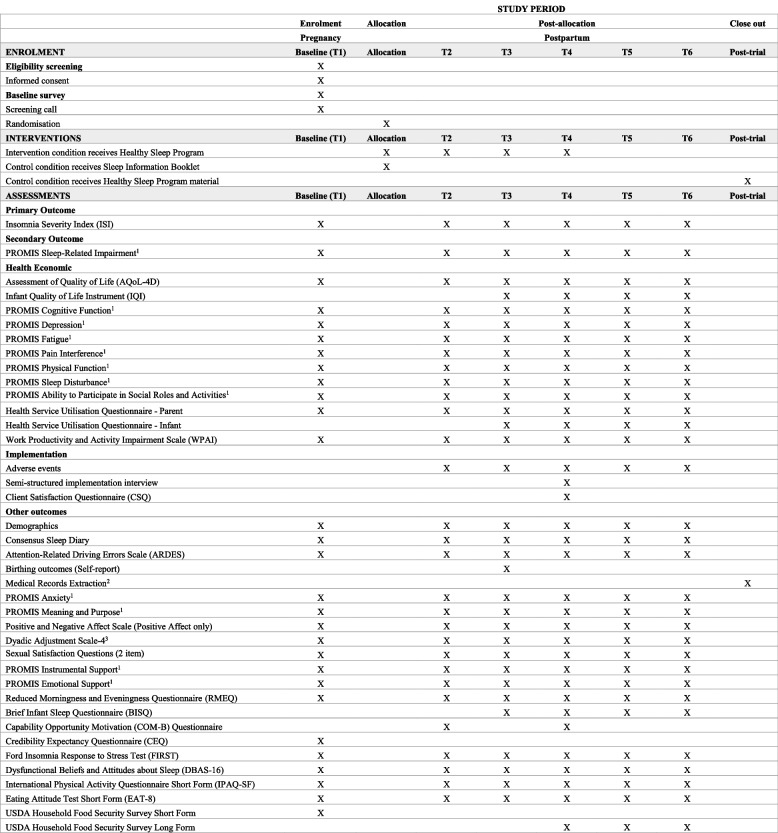
SPIRIT figure for schedule of enrolment, intervention, assessment and posttrial care for randomised controlled trial participants Note: T1, baseline (26–32 weeks pregnant); T2, 5 weeks post-intervention; T3, 3 months postpartum; T4, 6 months postpartum; T5, 12 months postpartum; T6, 24 months postpartum. X denotes a measure administered at that timepoint. ^1^*CAT*, computer adaptive testing version. ^2^Medical record extraction for participants from Monash Health or Royal Women’s Hospital who have consented to medical record being extracted. ^3^Presented to those who report currently in a relationship

Assessments of primary and exploratory outcomes and their timings are summarised in Fig. 2.

#### Primary outcome

The primary outcome for the effectiveness component is symptoms of insomnia, assessed using the ISI [26], a 7-item self-report measure rated on a 5-point Likert scale ranging from 0 to 4. Items are summed, and total scores range from 0 to 28. As long as four or more of the seven items are not missing for a participant at a specific timepoint, the ISI total score will be calculated using mean replacement, using the following formula, where *k* is the number of non-missing items:

$$ISI=\left(\frac{\sum_{i=1}^{k}{x}_{i}}{k}\right)7$$

There are two primary endpoints because sleep patterns differ substantially during pregnancy and postpartum. The pregnancy endpoint is the total continuous ISI score measured at 5 weeks after baseline (i.e. T2), comparing control to intervention group. The postpartum endpoint is the continuous average score of the non-missing ISI total scores across 3 (T3), 6 (T4), and 12 (T5) months postpartum representing overall symptom burden, comparing control to intervention group. For the postpartum endpoint, if the ISI is missing for two of the three timepoints, the endpoint will be considered missing.

#### Secondary outcomes

The secondary outcome for the effectiveness component of this trial of the present study is sleep-related impairment (SRI), measured using the Patient-Reported Outcomes Measurement Information System (PROMIS) Sleep-Related Impairment (computer adaptive test [CAT] Version) [30]. The SRI is automatically converted into T-scores and standard errors which have a general adult population mean of 50. As the SRI is measured using CAT, missing individual items are not allowed in Research Electronic Data Capture (REDCap) so it will either be complete or missing. Both pregnancy and postpartum endpoints will be applied for SRI in the same way as described for ISI above.

#### Health economic outcomes

The primary outcome for the economic evaluation will be total quality-adjusted life-years (QALYs) in the birthing parent *and* infant from baseline to 24 months postpartum based on the following:

1. *Parent quality of life*, as measured by total continuous QALYs from baseline (T1) to 24 months postpartum (T6), calculated based on Assessment of Quality of Life four dimension (AQoL-4D) index scores [31] in the main analysis. The AQoL-4D measures health-related quality of life on four dimensions: *independent living*, *social relationships*, *physical senses*, and *psychological well-being*
2. *Parent quality of life*, as measured by total continuous QALYs from baseline (T1) to 24 months postpartum (T6), calculated based on PROMIS-Preference (PROPr) scores [32] for sensitivity analysis. The PROPr reflects seven PROMIS domains: cognitive Function (CAT Version) [33], Depression (CAT Version) [34], Fatigue (CAT Version) [35], Pain Interference (CAT Version) [35], Physical Functioning (CAT Version) [35], Sleep Disturbance (CAT Version) [30] and the Ability to Participate in Social Roles (CAT Version) [35]
3. *Infant quality of life*, as measured by total QALYs from birth (T3) to 24 months (T6), calculated based on parent completion of the Infant Quality-of-Life Instrument (IQI) [36, 37]

The secondary outcome for the economic evaluation will be the primary clinical outcome during the postpartum period (average ISI during the postpartum period T3–T5), representing overall symptom burden for the birthing parent.

Results from the economic evaluation will be expressed as (1) cost per QALYs gained and (2) cost per point improvement on the primary clinical outcome where costs will encompass direct cost of the intervention, health service costs, and productivity costs as follows:

1. *Intervention costs* to 24 months postpartum for the *Healthy Sleep Program* and usual care
2. *Health service costs* from baseline to 24 months postpartum, calculated from parent self-report of health service utilisation for both birthing parent and infant
3. *Productivity costs* from baseline to 24 months postpartum, as measured by the Work and Productivity and Activity Impairment (WPAI) scale [38] for insomnia

#### Implementation outcomes

1. *Adverse events*, as measured by Adverse Event Checklist [39] at T2–T6.
2. *Barriers and enablers to sustained implementation*, explored through qualitative semi-structured interviews with treating clinicians, participants and their partners, and other relevant stakeholders (e.g. obstetricians and gynaecologists, general practitioners, midwives, psychologists) guided by the Theoretical Domains Framework (TDF) [40] and the Practical Robust Implementation and Sustainability Model (PRISM) [41]
3. *Satisfaction with intervention*, as measured by Client Satisfaction Questionnaire at T4 [42]

#### Exploratory outcomes

1. *Sleep duration, quality and characteristics*, measured using adapted Consensus Sleep Diary at T1–T6 [43]
2. *Driving impairment*, measured using Attention-Related Driving Errors Scale (ARDES) [44] at T1–T6
3. *Birthing and obstetric outcomes*, measured by self-report at T3 and by medical record extraction for participants who have provided consent and are patients at Monash Health or Royal Women’s Hospital
4. *Birthing parent mental health*, measured by PROMIS Anxiety (CAT Version) [34] at T1–T6, PROMIS Meaning and Purpose (CAT Version) [45] at T1–T6 and *positive affect*, as measured by Positive and Negative Affect Scale [46] at T1–T6
5. *Relationship satisfaction*, measured by brief Dyadic Adjustment Scale [47] for participants who have a current partner, at T1–T6
6. *Sexual Satisfactions*, as measured by questions “How often in the past 30 days have you engaged in any sexual activity (either with a partner or yourself)?” and “Over the past 30 days, how satisfied have you been with your sex life overall?”, adapted from PROMIS Sexual Function and Satisfaction [48], at T1–T6.

#### Other factors

The following are included to provide contextual information about the sample and for their potential role in treatment response:

1. *Demographic and medical conditions information* will be collected by self-report.
2. *Social support* as measured by PROMIS Instrumental & Emotional Support (CAT Version) [49] at T1–T6
3. *Chronotype* as measured by Reduced Morningness Eveningness Questionnaire (RMEQ) [50] at T1–T6
4. *Infant sleep (sleep onset latency, night-time awakening, daytime sleep duration, night-time sleep duration, nap duration,* etc.*)*, as measured by Brief Infant Sleep Questionnaire (BISQ) [51] at T3–T6
5. *Patient capability, opportunity and motivation for behaviour change*, as measured by brief Capability, Opportunity, Motivation - Behaviour (COM-B) questionnaire [52] at T2 and T4
6. *Patients’ perceived credibility and expectancy of treatment*, as measured by the Credibility Expectancy Questionnaire (CEQ) [53] at T1
7. *Stress vulnerability*, as measured by Ford Insomnia Response to Stress Test (FIRST) [54] at T1–T6
8. *Beliefs and attitudes about sleep*, as measured by Dysfunctional Beliefs and Attitudes about Sleep Scale (DBAS-16) at T1–T6 [55]
9. *Physical activity*, as measured by International Physical Activity Questionnaire Short Form (IPAQ-SF) [56] at T1–T6
10. *Attitude toward eating*, as measured by Eating Attitude Test Short Form (EAT-8) [57] at T1–T6
11. *Food security*, as measured by United States Department of Agriculture (USDA) Household Food Security Survey Short Form (T1) and Long Form (T4, T5, T6) [58]

### Harms {17}

Participants will complete an adverse event checklist [39] and report any hospitalisations at each timepoint following randomisation. Participants are also encouraged to contact the research team if any adverse events happen.

The following three levels of risk and adverse events are considered:

## Mental health-related risk

These are defined as intense psychological distress that may cause significant impairment to everyday functioning or impose risk to self or others (see Mental health risk protocol, supplementary material).

## Side effects

These are assessed at each post-baseline timepoint using an adverse events checklist for sleep interventions [39], which lists adverse effects that may (e.g. sleepiness) or may not (e.g. headaches) be the result of the intervention. Findings on these measures will be reviewed by the Data Safety Management Board (DSMB) at planned meetings. Participants will also be asked to contact the research team immediately if they experience any side effects that they may be attributing to the intervention.

## Severe adverse events

These are defined as maternal or infant deaths or serious complications that require hospitalisation (i.e. admission to ward). These events are reported at each assessment timepoint, and participants are encouraged to notify immediately if they have occurred. Expected events such as hospitalisation related to childbirth will be excluded from review.

Two investigators, one expert in sleep medicine and the other an obstetrician, will review all serious adverse events (SAEs) upon reporting and assess their expectedness and whether they may be related to the intervention. If any SAEs were suspected to be related to the intervention, the chair of the DSMB will be notified within 48 h. The chair will then decide whether an ad hoc meeting of the DSMB should be held. Harms will be analysed and reported in line with MedDRA structured vocabulary. Adverse events which are deemed to be potentially related to the intervention will be reported in trial publications.

### Participant timeline {18}

An overview of the participant timeline for those randomised in the trial is provided in Fig. 2.

### Sample size {19}

Sample size calculations for the present trial were informed by statistical, empirical, and clinical considerations, given cautions on basing power solely on pilot data [59]. Factors considered were as follows: (1) In a previous trial [21], Cohen’s d effect sizes for ISI and sleep-related impairment in women with baseline elevated ISI (> 7) ranged from 0.43 to 1.15. (2) Trial investigators with clinical experience discussed the smallest meaningful effect and identified a Cohen’s d = 0.40. (3) We calculated bootstrap confidence intervals for Cohen’s d from the previous trial data. Results showed that in > 75% of the time, the pilot effect size was Cohen’s d > 0.40, bolstering confidence that this clinically meaningful effect is highly plausible from our intervention.

For the effectiveness component of this trial, there are two primary outcomes (ISI at T2 and average ISI across T3–T5) and two secondary outcomes (SRI at T2 and average SRI across T3–T5) for a total of four hypothesis tests across primary and secondary outcomes. A Bonferroni correction for four tests would be *α* = 0.05/4 = 0.0125. We rounded down and conservatively set *α* = 0.01, two-tailed to adjust for multiple comparisons.

Planned primary and secondary analyses are four linear regression analyses, one for each outcome. Collectively, the models will have the following parameters estimated: 1 for the intercept, 1 for covarying for the outcome at baseline, 1 for the dummy-coded treatment condition, and 15 for the dummy-coded strata (see the “Sequence generation {21a} and {21b}” section for all 16 unique strata) resulting in a total of 18 estimated coefficients. For the pilot and for identifying a clinically important difference, we used Cohen’s d effect size. However, analyses are linear regressions to allow for covariates, so for power analyses, we translated Cohen’s *d* = 0.40 to a Cohen’s f2 of 0.04 using Cohen’s formula [60]:

$${f}^{2}={\left(\frac{d}{2}\right)}^{2}$$

We used the pwr.f2.test() function from the R package pwr to calculate the required sample size for the following conditions:

- Two-tailed *α* = 0.01.
- One numerator degree of freedom, testing the dummy-coded treatment condition
- 85% power
- 10% dropout (90% retained)
- 18 degrees of freedom used up in the regression models

A further sample size consideration is the stratified randomisation. We have 16 total strata (see the “Sequence generation {21a} and {21b}” section for details). A rule of thumb [61] for stratified randomisation using permuted blocks is that the number of strata should be no more than N/(4 × block size). As we are using a big stick design (BSD) for randomisation with maximally tolerated imbalance (MTI) = 2, the block size approach does not directly apply. However, a *MTI* = 2 for a two-group design yields the same possible maximum imbalance as permuted blocks with block size *B* = 4. Using this rule of thumb gives us a minimum required sample size to prevent overstratification of *N* = 16 × 16 = 256. Power analyses showed that a sample size of *N* = 384 provides ≥ 85% power to detect a Cohen’s *d* = 0.40 (Cohen’s f2 = 0.04) with two-tailed *α* = 0.01 to account for multiple comparisons and the outcome variable at baseline and all strata included as covariates. This also exceeds the minimum sample size needed for 16 strata. Thus, the final planned sample size was *N* = 384.

#### Updated increased sample size

When recruitment of the proposed sample size (*N* = 384) was complete, attrition and completeness of data were considered to assess the appropriateness of the sample size. Completion rates for T2, T3, and T4 varied between 74% and 84%, which was lower than the anticipated 90%. To account for this increased attrition, an increased sample size was required. The additional participant calculation is based on the following: If 70% of the sample completes at least 2 postpartum timepoints, a sample of 346/0.70 = 495 is needed; this is 111 more than planned. Thus, an increased proposed sample size of *N* = 495 is needed to account for potential impacts of attrition.

### Recruitment {20}

For public hospital recruitment, research midwives at Monash Health and the Royal Women’s Hospital will contact (via text message, phone call, e-mail, flyer, or in person at antenatal clinics) potentially eligible individuals. For online recruitment, online advertising will be conducted through social media and online newsletters. Recruitment will also be conducted in the community through study flyers in public areas (e.g. medical clinics and baby fairs).

When visiting the sign-up link, participants will be provided with a brief summary of information about the study. Participants are then able to assess their eligibility based on their gestation, age, and whether they live in Australia. If the potential participant is within 26–32 weeks of gestation, they will be invited to consent immediately; if a potential participant is less than 26 weeks of gestation, they will receive an e-mail inviting them to participate when they reach 26 weeks of gestation.

## Assignment of interventions: randomisation

### Sequence generation {21a} and {21b}

The random allocation sequence was developed by the trial statistician. Eligible participants will be randomised using a complete randomisation scheme generated in advance and conducted via REDCap [27, 28].

Randomisation will be stratified by the following:

1. Recruitment source (hospital patient coded as 0, other community recruitment coded as 1)
2. Insomnia severity (low < 14 coded as 0, high ≥ 14 coded as 1)
3. Gravidity (first pregnancy coded as 0, second or more coded as 1)
4. Baseline PROMIS depression symptoms (low ≤ 50 coded as 0, high > 50 coded as 1)

Fully crossed, this results in 2 × 2 × 2 × 2 = 16 unique strata. There are many algorithms to generate random sequences. Simple complete randomisation involves a fully random process, but this does not guarantee balance between the trial arms for a fixed sample size and may, by random chance, introduce time effects (e.g. theoretically it is possible, albeit unlikely, that the first 50% of participants are all randomised to intervention and the next 50% all to control). The permuted block randomisation (PBR) is perhaps the most common solution to this in clinical trials. PBR guarantees balance at the end of each block. However, the PBR introduces a new risk which is that it is predictable (e.g. in a block size of 4, if two people have been randomised to control, the researcher knows that the next two people will be randomised to intervention). BSD is another method of generating a random sequence that can be harder to guess and is theoretically superior to PBR [62]. To ensure a degree of balance, it is possible to specify the MTI. The MTI reflects the biggest sample size difference tolerated between conditions within the randomly generated sequence. We evaluated seven algorithms based on two properties: (1) The maximum imbalance observed during the sequence and (2) the probability of a correct guess, assuming the guess is based on whichever group is currently smaller. In both cases, lower is better reflecting less imbalance and a lower probability that the researcher may correctly guess, and thus bias, the next randomisation. We evaluated seven algorithms for randomisation using 10,000 randomly simulated sequences; these were as follows: (1) complete randomisation, (2) PBR, (3) random PBR (RPBR), (4) maximal procedure (MP), (5) BSD, (6) Efron’s biased coin design (EBCD), and (7) Chen’s design (CD). For each, we evaluated conditions with sequence length 100 and varying: (1) MTI of 2 and 4 for MP, BSD, and CD; (2) probability = 0.6 and 0.8 for EBCD and CD; (3) probability block size = 4, 6, and 8 for PBR; and (4) random blocks of size 4, 6, or 8 for RPBR. Results of these simulations revealed that for our purposes, the BSD with *MTI* = 2 provided optimal balance between minimising imbalance and reducing the probability that a researcher could correctly guess the next allocation. Therefore, we use BSD with *MTI* = 2.

### Allocation concealment mechanism {22}

Following confirmation of eligibility, the study staff will be able to randomise participants via the REDCap randomisation function. The randomisation allocation cannot be changed by staff either prior to or after randomisation. Staff will not be able to see the allocation sequence prior to randomisation being complete.

### Implementation {23}

The randomisation sequence will be built into REDCap [27, 28] by the trial statistician prior to the enrolment of the first participant. Randomisation will be conducted through REDCap by a trained research team member. The randomisation sequence cannot be accessed by staff members who are conducting the randomisation.

## Assignment of interventions: blinding

### Who will be blinded {24a}

Given the nature of the interventions, it is not possible for participants to be blind to their treatment allocation, but the condition pseudonyms “Group A” and “Group B” are used to avoid bias. As randomisation is completed by the research team and is necessary for administrative purposes, the study coordinator, research assistants, and clinicians will not be blinded to the group allocation. The chief investigator will be blinded to the group allocation except in exceptional circumstances where the risk to participants outweighs the benefit of blinding, such as evaluating a serious adverse event. Statisticians will not be blind to the condition in order to facilitate the conduction of statistical analysis.

### How will blinding be achieved {24b}

Individuals blinded to the intervention allocation (e.g. the chief investigator) will not have access to information related to participants’ group assignment, achieved via the role-based access in REDCap.

### Procedure for unblinding if needed {24c}

In the event of a serious adverse event, the research team will discuss the necessity, risk, and benefits of unblinding. If needed, the chief investigator will be unblinded, and the study coordinator will provide group allocation and other necessary information for investigation. No other participants’ allocation will be unblinded.

## Data collection and management

### Plans for assessment and collection of outcomes {25a}

Data will be collected, using validated questionnaires (see the “Outcomes {16}” section), via REDCap [27, 28] hosted on Monash University secure computing facilities. All questionnaires will be completed online via the REDCap system. Participants will be sent a unique participant access link to the questionnaires at the pre-specified assessment timepoints. To support the completeness of data, variables will be “required” to be entered by REDCap such that participants cannot move to the next page until all items are completed. Where data is missing or insufficient, the research team may contact the participant via email or phone to complete the remaining data. For participant screening calls, all researchers will be trained by MEC to promote reliability.

### Plans to promote participant retention and complete follow-up {25b}

Participants will receive at least three e-mail reminders to complete follow-up data collection. If no response is received via e-mail, at least one text message reminder will be sent. Participants will receive a gift card for each survey completed with $15AUD awarded for each of the first four surveys (T1, T2, T3, T4) and $20AUD for each of the last two surveys (T5, T6). Participants will also receive a study newsletter update at various points throughout the study to assist with retention.

### Data management {26}

Participants will complete online questionnaires through REDCap, hosted on Monash University secure computing facilities, with data backup handled by Monash University. Participant identifiable information (e.g. names, address) will only be stored in REDCap, which has role-based secure access and dual-authentication login. Outside REDCap participants will only be identified using a numeric ID. All interview and intervention data will be stored on a secure Monash server. The data collected will be retained for a minimum of 15 years. No more than 7 years after the publication, data will be de-identified, and the de-identified database will be made publicly available.

### Confidentiality {33}

Identifiable information will not be shared with anyone outside the research team except in the following context: (i) Participants have the right to access the information collected and stored by researchers about themselves; (ii) a research team member believes that there is a potential risk or danger posed to a participant or someone else, and their contact details may be provided to support services; or (iii) data may also be shared if required by law. All data is collected and stored in REDCap, hosted on Monash University secure computing facilities, with data backup handled by Monash University. Audio and video recordings will be stored on a secure Monash University drive, which can only be accessed by the research team using dual-authentication login. Identifying information will not be reported in publications.

## Statistical methods

### Statistical methods for primary and secondary outcomes {27a}, {27b} and {27c}

A detailed descriptive profile will be created for all outcomes, intervention conditions, sample characteristics, and intervention adherence. Descriptive statistics will be as follows: (1) For continuous variables that are approximately normally distributed without outliers: mean and standard deviation; (2) for continuous variables that are not normally distributed or have outliers: median and interquartile range; and (3) for discrete variables: frequency and percentage.

We will report the frequency and percent of participants who completed each timepoint and who have missing data at each timepoint. Adverse event frequency and type will be reported with frequencies and percentages. We will compare the proportion of withdrawn or missing participants at each timepoint and the proportion of adverse events by condition using chi-squared tests.

#### Effectiveness for Aim 1

To test aim 1, which is ISI (primary) and SRI (secondary) will be lower in the Healthy Sleep Program than the Control condition, we will use regression analyses. Analyses will be intention to treat, with participants analysed in the condition to which they were assigned regardless of actual intervention receipt. Significance is set at *α* = 0.01, two-tailed. We will calculate the expected raw difference in outcomes between treatment and control with 95% confidence intervals, along with the standardised difference to measure the effect size. All effectiveness analyses for Aim 1 will be conducted in R.

The dual primary (ISI at pregnancy and postpartum) and two secondary (SRI at pregnancy and postpartum) outcomes will be used as described previously (see “Outcomes {16}”) with the outcomes treated as continuous variables. Analyses will be separate regressions for each outcome, with the outcome as the dependent variable. Treatment condition will be dummy coded (Healthy Sleep Program = 1, sleep hygiene education = 0) and included as the focal predictor. The outcome measure at baseline (T1) will be included as a covariate. That is, ISI at T1 will be included for both ISI primary outcomes, and the SRI at T1 will be included for both SRI secondary outcomes. Randomisation strata will be fully crossed and dummy coded, and these dummy codes will be included as covariates [61]. The method selected for statistical inference for the main analyses will depend on features of the data.

If outcome data are missing, we will use multiple imputation through chained equations (MICE, [63]) based on predictive mean matching (PMM; [64]). We will generate 100 imputed datasets.

*Second*, if model residuals are approximately normally distributed, we will use normal theory-based standard errors to calculate confidence intervals and *p*-values. If model residuals are not normally distributed, we will use a nonparametric bootstrap with percentile-based confidence intervals based on 20,000 bootstrap samples. In this case, in addition to reporting 95% confidence intervals, we will use the 99% bootstrap percentile intervals to determine statistical significance based on whether or not the interval includes 0. The distribution of model residuals will be determined by fitting a linear regression model and creating a density plot of the residuals and a Q-Q plot of the observed residuals against a normal distribution.

*Third*, if there are no outliers in the model residuals, we will use regular linear regression. If there are outliers in the model residuals, we will use quantile regression and use a median (50th percentile) estimator. Outliers in the residuals will be determined by fitting a linear regression model, extracting the residuals and creating a density and rug plot.

The above choices will be combined as necessary based on the data. For example, if outcome data are missing, the residuals demonstrate non-normality and there are outliers, we would use quantile regression, non-parametric bootstrapping and multiple imputation. In cases where we use both bootstrapping and multiple imputation, we will use the approach of bootstrapping in the outer loop and then using imputation in the inner loop [65]. That is, for each of 20,000 bootstrap samples, we will use MICE with PMM to generate a single imputed dataset with added noise reflecting the uncertainty of the imputation, resulting in a total of 20,000 datasets that are both bootstrapped and imputed. This was selected as a simulation study demonstrated that while it is computationally demanding, it provides the best validity [65].

We will conduct and report sensitivity analyses. *First*, we will conduct a complier average causal effect analysis, which estimates the impact of an intervention in the population subgroup that complies with its assigned treatment, which will illuminate the intervention effect on women who received the minimum recommended amount of intervention. *Second*, we will conduct controlled multiple imputation, with an offset, δ, added to the imputed missing outcome data [66]. Specifically, we will vary *δ* assuming that participants who dropout and have missing data experience worse-than-expected outcomes. That is, a non-random pattern whereby people are more likely to dropout or have a missing outcome when the outcome is worse. We will vary the magnitude of *δ* and report treatment estimates across the range of *δ* values.

We will conduct two secondary analyses. First, we will test a condition × recruitment source interaction to assess intervention effect heterogeneity across sources. Second, we will conduct linear mixed models for ISI and SRI using scores at each individual timepoint to compare trajectories of insomnia and daytime symptoms between conditions. Protocol deviations will be documented, and sensitivity analyses will be carried out, if necessary, to examine whether findings hold with or without participants with deviations.

#### Economic evaluation for Aim 2

Cost-effectiveness and cost-utility analyses will be conducted from a societal perspective, summarising the health and productivity effects of the intervention during the trial period. Usual care is selected as the comparator to directly inform treatment and funding decisions. Direct costs of the active control condition will be excluded from analyses, with estimates of treatment effects for primary and secondary outcomes interpreted as conservative relative to usual care.

Primary and secondary outcomes for the economic evaluation are designed to capture hypothesised health gains during pregnancy for the pregnant participant and for the birthing parent and infant during the postpartum period. The primary outcome for the economic evaluation will be total QALYs in the birthing parent and infant from baseline to 24 months postpartum. For the birthing parent, total QALYs from baseline to 24 months postpartum will be calculated based on AQoL-4D [31] index scores at T1 to T6 in the main analysis and based on PROMIS-Pref (PROPr) index [32, 67] scores at T1 to T6 in sensitivity analyses. For infants, total QALYs from birth to 24 months will be calculated based on parent completion of the IQI at T3–T6 [36, 37]. The IQI is suitable for calculating QALYs in infants (0–12 months) [37], with coverage of seven domains of infant quality of life: sleeping, feeding, breathing, stooling/poo, mood, skin, and interaction.

The secondary outcome for the economic evaluation will be the primary clinical outcome during the postpartum period (average ISI during the postpartum period), representing overall symptom burden for the birthing parent and infant. Treatment effects with respect to QALYs will then be estimated using one-part generalised linear models (GLM), controlling for index scores at baseline and specifying appropriate variance and link functions [68].

Total cost per birthing parent–infant dyad will be calculated based on (i) per dyad cost of the intervention and usual care conditions, (ii) birthing parent self-report of health service utilisation for the birthing parent at T1 to T6 and for the infant at T3–T6 and (iii) productivity and activity impairment costs related to insomnia symptoms in the birthing parent during the trial period based on WPAI (insomnia) data at T1 to T6 [38]. Treatment effects with respect to total cost will be estimated using one-part GLM models with gamma variance function and a log link (rather than transformed ordinary least squares or two-part models [69]), controlling for birthing parent characteristics at baseline. Results will be expressed as (1) cost per QALY gained and (2) cost per point improvement on the primary clinical outcome. We will summarise sampling error and parameter uncertainty using the bootstrap acceptability method to calculate confidence intervals and generate cost-effectiveness acceptability curves [68].

#### Implementation evaluation for Aim 3

A subsample of randomised participants will be invited to take part in qualitative interviews to allow for exploration and analysis of the implementation potential of the Healthy Sleep Program. Additionally, clinicians who have delivered the intervention as well as perinatal professionals who routinely work with gestational parents (e.g. obstetricians and gynaecologists, general practitioners, midwives, psychologists) will be invited to participate in interviews.

The interviews will be semi-structured and aim to explore the barriers, enablers and contextual factors perceived to influence the sustainable implementation of the program. An interview guide with questions and prompts informed by the TDF [40] and PRISM [41] will guide the discussion. Interviews will be audio recorded and transcribed verbatim through a transcription service and/or by members of the research team. Transcripts will be imported into NVivo software [70] to manage the data and facilitate analysis. The interview transcripts with participants and their partners, treating clinicians and perinatal professionals will be analysed using thematic analysis to identify the factors perceived to influence the sustainable implementation of the Healthy Sleep Program. These factors will then be thematically mapped to the domains of the TDF and PRISM [71] to inform the design of implementation strategies to support wider scale-up and spread if shown to be clinically effective and cost-effective.

### Methods for additional analyses {27d}

Exploratory aims may be conducted via longitudinal analyses such as mixed effects models; latent growth models will be used to explore changes in outcomes over time, as well as predictors of change trajectories. Models such as cross-lagged panel analyses will be used to explore associations of constructs over time.

### Interim analyses {28b}

No interim analyses were planned, and no formal stopping rules are specified given the low risk of behavioural intervention used in this study, and that the benefits are unlikely to be overwhelmingly strong to warrant early termination of the trial.

### Protocol and statistical analysis plan {5}

The protocol and statistical analysis plan are included in the present manuscript.

## Oversight and monitoring

### Composition of the coordinating centre and trial steering committee {3d}

The steering committee will include the chief investigator, trial statistician, study coordinator, and site leads. Additionally, the study is also informed by the Community Reference Group which involves individuals with lived experience who provided insight prior to study commencement and will meet at least three times throughout the study. The study will be centrally coordinated from the Monash University site, with both Monash Health and the Royal Women’s Hospital sites having a site lead overseeing site-specific activities. The research team overseeing study operations (e.g. recruitment and intervention delivery) will meet weekly to assess progress. The investigators for the project will meet every 4 to 6 months to oversee the progress of the trial.

### Composition of the data monitoring committee and its role and reporting structure {28a}

The trial will be overseen by a DSMB which is responsible for the stewardship of the trial over all participating sites or institutions. The stewardship will include continuous review of participant recruitment, accrual, retention, and withdrawal. It further involves oversight of participant management, adherence to protocol-specified regimens, and procedures for data management and quality control. The DSMB will involve a minimum of four independent (i.e. not directly involved in trial) experts in the areas of obstetrics and sleep health. The DSMB will be responsible for safeguarding the interests of trial participants by assessing the safety of the interventions during the trial and the general progress of the trial. All DSMB members will be independent of the funder.

### Frequency and procedures for monitoring trial conduct {29}

The trial will be overseen by DSMB. The DSMB will be presented with a report of data completeness, ongoing recruitment status (e.g. target recruits, participants recruited, timeliness) and intervention adherence. The DSMB will meet at least three times during the trial, with the chair of DSMB to call ad hoc meetings as needed.

### Protocol amendments {31}

If protocol amendments are required, these amendments will be submitted to the primary ethics committee (Monash Health Human Research Ethics) and communicated to all other ethics committees following approval. If these amendments will impact existing participants, these participants will be contacted via email or phone. Protocol amendments will be reported in publications.

## Dissemination policy {8}

Irrespective of study outcomes, results of the study will be disseminated through publication of peer-reviewed journal articles and through conference presentations. Results of the study may also contribute to theses of research students. Participants will be sent a summary of the study outcome about 18 months after the completion of the study. If shown clinically effective and cost-effective, the research team plans to work with clinical partners to implement the intervention in community perinatal care. Authorship for dissemination publications will be decided by the International Committee of Medical Journal Editors’ Authorship guidelines.

## Discussion

This project will provide needed empirical data on the effectiveness, cost-effectiveness and implementation potential of a cognitive behavioural therapy for insomnia in the perinatal period. Importantly, this study will inform potential future implementation of CBT-I for perinatal insomnia symptoms in community care by identifying barriers and enablers to sustainable implementation of a scalable CBT-I program for perinatal populations. Additionally, through the implementation assessment, this study will identify ways in which (i.e. through midwives, hospitals, general practitioners (GP) clinics) at-risk perinatal individuals may be identified and targeted for treatment.

The results of the wider implementation of this intervention may improve future sleep health and mental health outcomes for individuals during the perinatal period, thus reducing the excessive burden of health care providers and improving outcomes for individuals and families.

## Trial status

Protocol Version 5, dated 29 September 2025. Participant recruitment began on 09/02/2023, with the first participant enrolled on 20/02/2023. Recruitment closed after protocol submission (07/10/2024) on 01/12/2024, with the final participant enrolled on 08/11/2024. The trial data collection is ongoing with the last planned follow-up to be conducted on approximately 01/03/2027.

## Supplementary Information

Supplementary Material 1. Auxiliary studies: 1. Observational longitudinal study of birthing parents with low insomnia and 2. Observational longitudinal study of Partners of pregnant individuals. Mental health risk protocol. SHINE Participant information sheet and consent form.

## Data Availability

After completion of the study, no more than 7 years after publication, data will be de-identified and made publicly available through Monash bridges (or a similar data repository). Statistical analysis code will be published via journal publications or public code sharing repositories (e.g. GitHub).

## Abbreviations

ARDES: Attention-Related Driving Errors Scale
AQoL-4D: Assessment of Quality of Life
AUD: Australian Dollar
BISQ: Brief Infant Sleep Questionnaire
BSD: Big Stick Design
CAT: Computer Adaptive Testing
CBT-I: Cognitive Behavioural Therapy for Insomnia
CD: Chen’s Design
CEQ: Credibility Expectancy Questionnaire
COM-B: Capability, Opportunity, and Motivation - Behaviour
DBAS-16: Dysfunctional Beliefs and Attitudes Scale
DSMB: Data Safety Management Board
EAT-8: Eating Attitude Test Short Form
EBCD: Efron’s Biased Coin Design
FIRST: Ford Insomnia Response to Stress Test
GLM: Generalized Linear Models
GP: General Practitioner
IPAQ-SF: International Physical Activity Questionnaire-Short Form
IQI: Infant Quality of Life Instrument
ISI: Insomnia Severity Index
MICE: Multiple Imputation through Chained Equations
MP: Maximal Procedure
MTI: Maximally Tolerated Imbalance
PBR: Permuted Block Randomisation
PICF: Participant Information and Consent Form
PMM: Predictive Mean Matching
PRISM: Practical Robust Implementation and Sustainability Model
PROMIS: Patient Reported Outcomes Measurement Information System
PROPr: Patient Reported Outcomes Measurement Information System — Preference
QALY: Quality-Adjusted Life Years
RCT: Randomised Controlled Trial
REDCap: Research Electronic Data Capture
RMEQ: Reduced Morningness-Eveningness Questionnaire
RPBR: Random Permuted Block Randomisation
SAE: Serious Adverse Events
SHINE: Sleep Health in Perinatal Care
SRI: Sleep-Related Impairment
TDF: Theoretical Domains Framework
USDA: United States Department of Agriculture
WPAI: Work and Productivity and Activity Impairment
