# Supplementary material for: A scalable cognitive behavioural therapy intervention for perinatal insomnia: a protocol for a hybrid effectiveness-implementation type 1 randomised controlled trial
Source: Trials. 2025 Dec 30;27:181. doi: 10.1186/s13063-025-09308-5 (PMC12934060; doi:10.1186/s13063-025-09308-5)
Supplement: Supplementary file 1 — Supplementary Material 1. Auxiliary studies: 1. Observational longitudinal study of birthing parents with low insomnia and 2. Observational longitudinal study of Partners of pregnant individuals. Mental health risk protocol. SHINE Participant information sheet and consent form. [file 13063_2025_9308_MOESM1_ESM.docx]

# Title

A scalable cognitive behavioural therapy intervention for perinatal insomnia: A protocol for a hybrid effectiveness-implementation type 1 randomised controlled trial

**Authors:** Meagan E Crowther^1^, Orly Atzmon^1^, Christie J Bennett^2^, Margot Davey^3^, Sean P A Drummond^1^, Rachel Manber^4^, Ben W Mol^5^, Duncan Mortimer^6^, Denise A O’Connor^7^, Daniel L Rolnik^5^, Jenny Ryan^8^, Joshua F Wiley^1^, Bei Bei^1*^

^1^ School of Psychological Sciences, Faculty of Medicine, Nursing and Health Sciences, Monash University, Clayton Victoria Australia
2 Department of Nutrition, Dietetics and Food, School of Clinical Sciences, Monash University, Clayton Victoria, Australia
3 Melbourne Children's Sleep Centre, Monash Children’s Hospital, Monash Health, Clayton Victoria, Australia
4 Department of Psychiatry and Behavioral Sciences, School of Medicine, Stanford University, California, USA
5 Department of Obstetrics and Gynaecology, Monash University, Clayton Victoria, Australia
6 Centre for Health Economics, Monash University, Caulfield East Victoria, Australia
7 School of Public Health and Preventive Medicine, Monash University, Melbourne Victoria, Australia
8 Royal Women's Hospital, Parkville Victoria, Australia

***Corresponding Author**: A/Prof Bei Bei**:** bei.bei@monash.edu

# Supplementary materials

1. Auxiliary studies: 1. Observational longitudinal study of birthing parents with low insomnia and, 2. Observational longitudinal study of Partners of pregnant individuals.
2. Mental health risk protocol
3. SHINE Participant information sheet and consent form.

# Auxiliary Study: Longitudinal observational study.

## Background

Sleep disturbance is prevalent during the perinatal period, with about 70% of individuals experiencing sleep disturbance during pregnancy [1], and a similar proportion (66%) of birthing parents continuing to experience sleep complaints well into the first year postpartum [1]. To date, there is limited understanding of what factors contribute to the emergence of parental sleep problems during early parenthood, and little consideration of perinatal sleep disturbance in fathers and partners [2]. Therefore, there is a need for studies which examine i) those that current experience low sleep complaints during pregnancy and ii) partners of pregnant individuals.

## Objectives

**Aim 1.** An exploratory investigation into the course and risk/protective factors for the development of new-onset insomnia symptoms during the perinatal periods, to inform future insomnia prevention efforts. This requires longitudinal observations of individuals with low insomnia symptoms at baseline.

**Aim 2**. An exploratory investigation into the changes in, and predictors of, sleep, insomnia symptoms, and wellbeing of partners of pregnant individuals. This requires longitudinal observations of partners of pregnant individuals.

**Study design**

Longitudinal observation of cohorts who did not receive intervention (for Aims 1-2).

## Low Sleep Complaint Observational Cohort

For Aim 1, we will conduct longitudinal observations on pregnant individuals who score ≤ 7 on the ISI and thus were not eligible for the RCT. Eligibility for the observational cohort is:

1. Aged 18 years or older
2. At least 26 weeks but no more than 32 weeks of gestation
3. Able to communicate in English
4. Have regular access to e-mail and the internet
5. Currently living in Australia

There are no additional exclusion criteria for observational birthing participants. Participants who are not eligible for either the RCT or the Intervention-Only Cohort will also be invited to this cohort.

### Partners Observational Cohort

For Aim 1, the study will involve longitudinal observations of partners of pregnant individual. Inclusion criteria for this cohort is:

1. Aged 18 years or older
2. Partner is at least 26 weeks but no more than 32 weeks of gestation
3. Able to communicate in English
4. Have regular access to e-mail and the internet
5. Currently living in Australia

The pregnant individual does not need to be part of one of the auxiliary trials for the partner to take part.

## Outcomes

An overview of participant timeline and outcomes for specific participants groups for those in the auxiliary studies is provided in Figure S1. Not all measures presented below are completed by both groups (see Figure S1).

- - - 1. *Insomnia symptoms*, as measured by Insomnia Severity Index [3]

1. *Sleep Disturbance*, as measured by Sleep Disturbance (CAT Version) [4]
2. *Sleep-related impairment,* measured using the PROMIS Sleep Related Impairment (CAT Version) [4].
3. *Sleep duration, quality and characteristics,* measured using adapted Consensus Sleep Diary [5].
4. *Driving impairment,* measured using Attention-Related Driving Errors Scale (English) [6].
5. *Birthing and obstetric outcomes (Birthing parent only),* measured by self-report at T3 and by medical record extraction in participants from Monash Health and Royal Women’s Hospital.
6. *Mental health,* measured by PROMIS Anxiety (CAT Version) [7], PROMIS Meaning and Purpose (CAT Version) [8], *Positive Affect,* as measured by Positive and Negative Affect Scale [9].
7. *Relationship Satisfaction,* measured by brief Dyadic Adjustment Scale [10] for participants who have a current partner.
8. *Sexual Satisfaction,* as measured by questions “How often in the past 30 days have you engaged in any sexual activity (either with a partner or yourself)?” and “Over the past 30 days, how satisfied have you been with your sex life overall?” these questions were adapted by PROMIS Sexual Function and Satisfaction [11].
9. *Attachment to infant (Non-birthing only)*, as measured by Paternal Antenatal Attachment Scale (Pregnancy) and Paternal Postnatal Attachment Scale (Postpartum).
10. Demographic and medical conditions information will be collected by self-report.
11. Social support as measured by PROMIS Instrumental Emotional Support (CAT Version) [12].
12. Chronotype as measured by Reduced Morningness Eveningness Questionnaire (RMEQ) [13].
13. *Parent reported infant sleep,* as measured by Brief Infant Sleep Questionnaire (BISQ) [14].
14. *Stress vulnerability*, as measured by Ford Insomnia Response to Stress Test (FIRST) [15].
15. *Beliefs and attitudes about sleep,* as measured by Dysfunctional Beliefs and Attitudes Scale (DBAS-16) [16].
16. *Physical Activity,* as measured by International Physical Activity Questionnaire Short Form (IPAQ-SF) [17].
17. *Attitude toward eating,* as measured by Eating Attitude Test Short Form (EAT8) [18].
18. *Food Security,* as measured by United States Department of Agriculture (USDA) Household Food Security Survey Short Form (T1) and Long Form (T3, T4, T5) [19].

|  | **Enrolment** | **Follow-up** | | | | | **Notes** |
| --- | --- | --- | --- | --- | --- | --- | --- |
|  | **Pregnancy** | | **Postpartum** | | | |  |
| **ENROLMENT** | **Baseline (T1)** | **T2** | **T3** | **T4** | **T5** | **T6** |  |
| **Eligibility screening** | X |  |  |  |  |  |  |
| Informed consent | X |  |  |  |  |  |  |
| **ASSESSMENTS** | **Baseline (T1)** | **T2** | **T3** | **T4** | **T5** | **T6** |  |
| Insomnia Severity Index (ISI) | X | X | X | X | X | X |  |
| Demographics | X | X | X | X | X | X |  |
| PROMIS Cognitive Function | X | X | X | X | X | X |  |
| PROMIS Depression | X | X | X | X | X | X |  |
| PROMIS Fatigue | X | X | X | X | X | X |  |
| PROMIS Sleep Disturbance | X | X | X | X | X | X |  |
| Health Service Utilisation Questionnaire - Parent | X | X | X | X | X | X | Birthing only |
| PROMIS Sleep related impairment^1^ | X | X | X | X | X | X |  |
| Consensus Sleep Diary | X | X | X | X | X | X |  |
| Attention-Related Driving Errors Scale | X | X | X | X | X | X |  |
| PROMIS Anxiety^1^ | X | X | X | X | X | X |  |
| PROMIS Meaning and Purpose^1^ | X | X | X | X | X | X |  |
| Positive and Negative Affect Scale (Positive Affect) | X | X | X | X | X | X |  |
| Dyadic Adjustment Scale-4^3^ | X | X | X | X | X | X |  |
| Sexual Satisfaction Questions (2 item) | X | X | X | X | X | X |  |
| PROMIS Instrumental Support^1^ | X | X | X | X | X | X |  |
| PROMIS Emotional Support^1^ | X | X | X | X | X | X |  |
| Reduced Morningness and Eveningness Questionnaire | X | X | X | X | X | X |  |
| Ford Insomnia Response to Stress Test (FIRST) | X | X | X | X | X | X |  |
| Dysfunctional Beliefs and Attitudes About Sleep (DBAS-16) | X | X | X | X | X | X |  |
| International Physical Activity Questionnaire Short Form (IPAQ-SF) | X | X | X | X | X | X |  |
| Eating Attitude Test Short Form - EAT8 | X | X | X | X | X | X |  |
| USDA Household Food Security Survey Short Form | X |  |  |  |  |  |  |
| USDA Household Food Security Survey Long Form |  |  |  | X | X | X |  |
| Birthing outcomes (Self-report) |  |  |  | X |  |  | Birthing only |
| Medical Records Extraction^2^ |  |  |  |  |  | X | Birthing only |
| Paternal Antenatal Attachment Scale | X | X |  |  |  |  | Non-birthing only |
| Paternal Postnatal Attachment Scale |  |  | X | X | X | X | Non-birthing only |
| Infant Quality of Life Instrument (IQI) |  |  | X | X | X | X | Birthing only |
| Brief Infant Sleep Questionnaire |  |  | X | X | X | X |  |
| Health Service Utilisation Questionnaire - Infant |  |  | X | X | X | X | Birthing only |

Figure S1. Schedule of assessments for auxiliary study participants.

Note. T1 = Baseline (26-32 weeks pregnant), T2 = 5 weeks post baseline, T3 = 3 months postpartum, T4 = 6 months postpartum, T5 = 12 months postpartum, T6 = 24 months postpartum. X denotes a measure administered at that time point, ^1^CAT: Computer Adaptative Testing Version, , ^2^Medical record extraction for participants from Monash Health or Royal Women’s Hospital who have consented to medical record being extracted, ^3^Presented to those who report currently in relationship

# Sample size

The present study is proposed as an auxiliary exploratory study and thus no formal sample size calculation has been undertaken. Recruitment for study will continue in line with RCT until such time as an appropriate RCT sample has been reached.

# Recruitment

Recruitment will be carried out simultaneously with the RCT using the same methods described in the main paper.

# Data collection and management

## Plans for assessment and collection of outcomes

See Outcomes section.

## Plans to promote participant retention and complete follow-up

Participants will receive multiple e-mail reminders to complete follow-up data collection. If no response received via e-mail, text message reminders will be sent. Participants will receive a gift card for each survey completed with $15AUD awarded for each of the first four surveys (T1, T2, T3, T4) and $20AUD for each of the last two surveys (T5, T6). Participants will also receive a study newsletter update at various points throughout study to assist with retention.

## Data management and Confidentiality

See main paper text.

**Statistical Methods**

Changes in sleep and mental health outcomes will be conducted via longitudinal analyses such as mixed effects models, latent growth models will be used to explore changes in outcomes over time, as well as predictors of change trajectories. Models such as cross-lagged panel analyses will be used to explore associations of constructs over time.

**Dissemination plans**

See main paper text.

**Discussion**

The results from the observational arms will begin to address the dearth of evidence regarding development of and contributors to poor sleep and mental health symptoms in early parenthood. In particular, the study will provide evidence on the potential longitudinal changes in sleep and wellbeing in fathers and non-birthing parents. These findings will provide evidence regarding risk factors associated with the development of sleep complainants during pregnancy and postpartum.

# References for Auxiliary studies

1. Lee KA. Alterations in sleep during pregnancy and postpartum: a review of 30 years of research. Sleep Med Rev. 1998; doi: 10.1016/s1087-0792(98)90010-7.

2. Wynter K, Francis LM, Fletcher R, McBride N, Dowse E, Wilson N, et al. Sleep, mental health and wellbeing among fathers of infants up to one year postpartum: A scoping review. Midwifery. 2020; doi: 10.1016/j.midw.2020.102738.

3. Bastien CH, Vallières A, Morin CM. Validation of the Insomnia Severity Index as an outcome measure for insomnia research. Sleep Med. 2001; doi:10.1016/s1389-9457(00)00065-4.

4. Buysse DJ, Yu L, Moul DE, Germain A, Stover A, Dodds NE, et al. Development and Validation of Patient-Reported Outcome Measures for Sleep Disturbance and Sleep-Related Impairments. Sleep. 2010;33:781–92.

5. Carney CE, Buysse DJ, Ancoli-Israel S, Edinger JD, Krystal AD, Lichstein KL, et al. The consensus sleep diary: standardizing prospective sleep self-monitoring. Sleep. 2012; doi: 10.5665/sleep.1642.

6. Barragán D, Roberts DM, Baldwin CL. Validation of the Attention-Related Driving Errors Scale (ARDES) in an English-speaking sample. Hum Factors. 2016; doi: 10.1177/0018720816657927.

7. Pilkonis PA, Choi SW, Reise SP, Stover AM, Riley WT, Cella D, et al. Item banks for measuring emotional distress from the Patient-Reported Outcomes Measurement Information System (PROMIS®): depression, anxiety, and anger. Assessment. 2011; doi: 10.1177/1073191111411667.

8. Salsman JM, Lai J-S, Hendrie HC, Butt Z, Zill N, Pilkonis PA, et al. Assessing psychological well-being: self-report instruments for the NIH Toolbox. Qual Life Res. 2014; doi: 10.1007/s11136-013-0452-3.

9. Watson D, Clark LA, Tellegen A. Development and validation of brief measures of positive and negative affect: the PANAS scales. J Pers Soc Psychol. 1988; doi: 10.1037//0022-3514.54.6.1063.

10. Sabourin S, Valois P, Lussier Y. Development and validation of a brief version of the dyadic adjustment scale with a nonparametric item analysis model. Psychol Assess. 2005; 10.1037/1040-3590.17.1.15.

11. Weinfurt KP, Lin L, Bruner DW, Cyranowski JM, Dombeck CB, Hahn EA, et al. Development and Initial Validation of the PROMIS® Sexual Function and Satisfaction Measures Version 2.0. J Sex Med. 2015; doi: 10.1111/jsm.12966.

12. Hahn EA, DeWalt DA, Bode RK, Garcia SF, DeVellis RF, Correia H, et al. New English and Spanish social health measures will facilitate evaluating health determinants. Health Psychol. 2014; doi: 10.1037/hea0000055.

13. Adan A, Almirall H. Horne & Östberg morningness-eveningness questionnaire: A reduced scale. Pers Individ Dif. 1991; doi: 10.1016/0191-8869(91)90110-W.

14. Sadeh A. A brief screening questionnaire for infant sleep problems: validation and findings for an Internet sample. Pediatrics. 2004;doi: 10.1542/peds.113.6.e570.

15. Drake C, Richardson G, Roehrs T, Scofield H, Roth T. Vulnerability to stress-related sleep disturbance and hyperarousal. Sleep. 2004; doi: 10.1093/sleep/27.2.285.

16. Morin CM, Vallières A, Ivers H. Dysfunctional Beliefs and Attitudes about Sleep (DBAS): Validation of a Brief Version (DBAS-16). Sleep. 2007; doi: 10.1093/sleep/30.11.1547.

17. Craig CL, Marshall AL, Sjöström M, Bauman AE, Booth ML, Ainsworth BE, et al. International physical activity questionnaire: 12-country reliability and validity. Med Sci Sports Exerc. 2003; doi: 10.1249/01.MSS.0000078924.61453.FB.

18. Richter F, Strauss B, Braehler E, Altmann U, Berger U. Psychometric properties of a short version of the Eating Attitudes Test (EAT-8) in a German representative sample. Eat Behav. 2016; doi: 10.1016/j.eatbeh.2016.03.006.

19. United States Department of Agriculture, Economic Research Service. U.S. Household Food Security Survey Module [Internet]. Washington (DC): USDA ERS; 2025. Available from: *https://www.ers.usda.gov/topics/food-nutrition-assistance/food-security-in-the-us/survey-tools*

# SHINE Mental health risk protocol

***Purpose***

To uphold the duty of care for all participants and make appropriate referrals for those who pose a risk of harm to self or others.

***Procedure***

All participants: For all participants, if they score 1 standard deviation above the population mean on PROMIS Depression or Anxiety scales, at any of the assessment time point, they will be sent the list of support services and encouraged to seek further support.

***Potential intervention participants***

***Step 1:*** A brief risk assessment will be conducted in all participants who scored > 7 on the Insomnia Severity Index (i.e., potential participants for intervention randomisation) by a research team member under supervision of a Clinical Psychologist during telephone screening. Participants will be told that we ask everyone these questions, not just themselves (e.g., "I am now going to ask you a few questions which may be hard to talk about, but it’s important for us to know and we ask these to absolutely everyone who takes part in the project". The brief risk assessment will assess: (a) *Ideation*: suicidal ideation, or thoughts of harm to self/others (e.g., Do you currently have thoughts of harming yourself or ending your life?); (b) *Plans*: of suicide or harm to self/others (e.g., Do you have a plan for how you may harm yourself?); (c) *Previous attempt(s)*: prior attempts or harm to self/others (e.g., Have you attempted suicide/self-harm before?); (d) *Presence of current mental health care* and whether provider(s) aware of risks if any (e.g., Are you currently receiving support from a mental health professional regarding these thoughts?).

***Step 2:*** If a participant discloses (a) ideation regarding harm to self/others, check whether the participant endorses (b) plans of suicide or harm to self/others, and (c) previous attempts. Next, check if participant is engaged in mental health care.

-> If YES: (a) Remind participant that the Participant Information and Consent Form asked for consent to contact healthcare providers in case of heightened risk during project participation (e.g., disclosure of harm to self/others); (b) Ask participant for the contact details of their mental health care provider, explaining that these details are stored confidentially and will enable our team to liaise with providers and tailor care, if needed; (c) Encourage participant to continue engaging in treatment with provider. Go to Step 3.

-> If NO: Go to Step 3

***Step 3:*** Decide on appropriate action.

**Low Risk**: (a) Denies ideation; (b) Denies plan.

*Action* -> Eligible to participate if no other exclusion criteria are met.

**Medium Risk**:(a) Discloses ideation BUT (b) Denies plan

*Action* -> (a) If participant is not currently engaged in treatment, encourage them to seek support. Discuss accessing mental health care via GP (e.g., Better Access to Mental Health Care Initiative). Provide contact details of mental health services below (includes 24-hour help lines), (b) Discuss case with Clinical Lead and/or Chief Investigator; document all communications, (c) If help-seeking and willingness to participate, include in study if no other exclusion criteria met.

**High Risk:** (a) Discloses BOTH ideation and plan, OR (b) Discloses ideation and previous attempt(s), denies plan.
*Action* -> Exclude from study and do the following: (a) Confirm address/contact of participant., (b) Call team clinician on-call and discuss most appropriate course of action case-by-case, (c) Encourage participants to engage with mental health service as a priority (rather than participating in research); they are welcome to re-engage with the study once mental health support is in place, (d) If participant re-engages, repeat risk assessment. Participant could participate if risk status has reduced to 'low' or 'medium', (e) Consult Clinical Lead and/or Chief Investigator along the way and document all communications.

***Step 4:*** Make follow-up contact (within 24 hrs to 1 week depending on urgency) for those who pose a medium/high risk. Confirm they have sought support.

# SHINE Participant information sheet and consent form

## SHINE Participant Information Sheet - Mothers and Birthing Parents

**Title:** SHINE: Sleep Health in Perinatal Care

**Investigators:** Assoc Prof Bei Bei (primary contact), Prof Rachel Manber, Prof Sean Drummond, Dr Joshua Wiley, Assoc Prof Duncan Mortimer, Assoc Prof Denise O'Connor, Prof Ben W. Mol, Assoc Prof Daniel Rolnik, Assoc Prof Margot Davey, Dr Meagan Crowther, Dr Nina Quin, Dr Christie Bennett, Ms Jenny Ryan

**Project Sponsor:** Monash University

**Location:** Online and remote participation.

**Recruitment sites:** Monash Health sites, Royal Women's Hospital, Community.

**1. Introduction**

This information sheet is for mothers and birthing parents. The SHINE Project also enrols fathers and non-birthing parents. If your partner would like to participate, they can sign up on this same link: SIGN UP LINK

You are invited to take part in this research project because you are expecting a baby. This Participant Information Sheet/Consent Form tells you about the research project. It explains the tests and intervention involved. Knowing what is involved will help you decide if you want to take part in the research. Please read this information carefully. Ask questions about anything that you don't understand or want to know more about. Before deciding whether or not to take part, you might want to talk about it with a relative, friend or your local doctor.

You will be emailed a copy of this Participant Information and Consent Form to keep.

**2. What is the purpose of the research?**

Expecting and welcoming a new child is associated with changing sleep patterns. Some of these changes to sleep can be challenging. The SHINE project is funded by the Australian government to (1) better understand sleep and wellbeing of new parents during pregnancy and the postnatal period, and (2) test interventions that could eventually be used in perinatal care to support healthy sleep in the perinatal periods. This research has been funded by National Health and Medical Research Council.

**3. Who can take part in the research?**

Individuals meeting the following criteria are welcome to sign up:

Aged 18 years or older.

In 26 to 32 weeks gestation of pregnancy. If you are currently less than 26 weeks pregnant, you are welcome to sign up, and we will contact you when you reach 26 weeks.

Able to communicate in English.

Have regular access to email and the internet. Currently living in Australia.

You do not need to have sleeping difficulties to sign up and participate.

Mothers and birthing parents who currently experience sleeping difficulties, along with their partners or other carers of the infant (if choose to), may be offered one of two sleep intervention programs.

There is no cost to participate in this study.

**4. What does participation involve?**

All participation can be completed online, via telephone or telehealth.

**SIGN UP**

If you meet above criteria (3. Who can take part in research?) and complete the consent form, we will ask you to answer a few questions about your sleep, mental health, and physical health (takes ~2 minutes). After this brief sign-up, if the project is suitable for you, we will welcome you to complete the first survey. Before deciding whether you will be assigned to one of the sleep programs, a researcher may contact you and seek clarification about the answers you provided.

**ALL PARTICIPANTS**


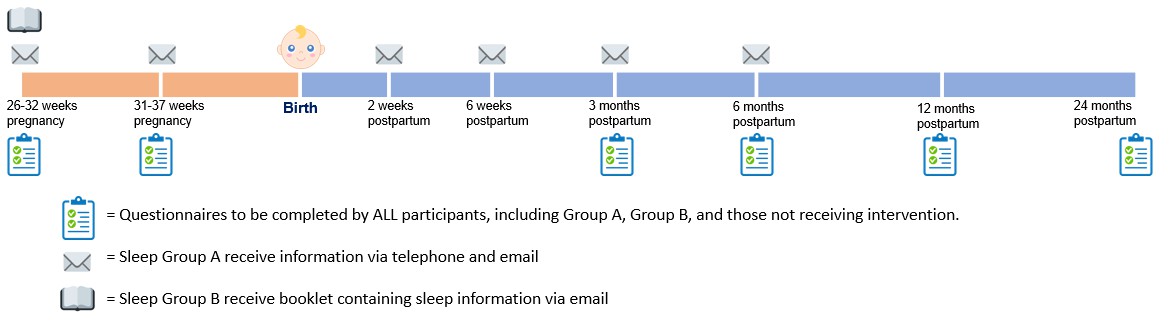


**Questionnaires:** All participants will be asked to fill in online questionnaires once at each of the following times. The questionnaires will take about 30 minutes at each time.

**1.** At the enrolment;

**2.** 5 weeks after enrolment;

**3.** When your child is 3 months old;

**4.** When your child is 6 months old;

**5.** When your child is 1 year old;

**6.** When your child is 2 years old.

The questionnaires will ask about a range of experiences such as your sleep, physical and mental health, access to care, as well as your infant's health and your experience as a parent after they are born.

**Medical Records:** If you give birth at Monash Health or the Royal Women's Hospital, we will also ask your permission to collect some information from your medical record (such as your health during pregnancy and birth, your baby's weight, length etc.). If you do not wish for your medical records to be extracted, or only certain information to be extracted, this will not affect your eligibility for this project. For details on what information can be collected, please see the consent form.

**Token of appreciation:** As a token of our appreciation for your participation in this project, we will send you a $15 digital gift vouchers for each of the first 4 surveys, and a $20 voucher for the last two surveys, up to $100 in total. If your partner also participates in SHINE, they will receive a separate voucher for their own participation.

**Future studies:** When participating in SHINE, we may contact you for a small number of related studies. After completing the last questionnaire, we may also contact you to extend your participation so we can better understand sleep and wellbeing beyond the first 2 years' of new parenthood.

**SLEEP INTERVENTION (a sub-group of participants)**

Participants who experience sleeping difficulties at enrolment may be allocated to one of the two sleep programs. If you are selected to take part in one of the intervention groups, it will involve:

**Group A:** Participants in this group will receive information and strategies to improve sleep during pregnancy and the postpartum periods. This includes:

**1.** Telephone or telehealth consultations with a registered or provisional psychologist:

- 1 full consultation (~60 minutes) at enrolment;

- Up to 3 mini-consultations (~15 minutes) before childbirth;

- 1 mini-consultation (~ 30 minutes) when the newborn is 3 months.

- Partner or another person who will also be involved in caring for the newborn is encouraged to attend at least one of the above consultations.

**2.** Digital materials on healthy sleep strategies during pregnancy, as well at 2 weeks, 1.5 months, 3 months, and 6 months after childbirth.

Each of the digital materials will take no longer than 10 minutes to read. At each time there will be between 3 to 6 materials (30min to 60min to read/listen to in total). The materials will be delivered to you every few days during the period, so you can digest them one at a time.

The telephone or telehealth consultations will be conducted with a trained researcher and all telephone communications will be audio recorded. Telephone and telehealth calls are recorded for training and quality assurance purposes.

**Group B:** Participants in this group will receive a booklet containing sleep information and sleep hygiene relevant to the pregnancy and postpartum period. This will be delivered electronically during pregnancy for use throughout the pregnancy and postpartum period. The sleep information booklet will take approximately 15 minutes to read.

**Both Group A and B**: After signing up to the study, if you are allocated to receive one of the two sleep programs, a researcher will contact you and let you know the next steps. We will also discuss with you whether someone else (e.g., partner, family members) wish to support your involvement in the sleep program, for example, both receiving the digital materials to be on the same page, join some/part of the consultations with you. After completing the final survey at 2 years postpartum, we will send you the digital intervention materials received by the group you were not allocated to.

Evaluating the programs. Participants in the sleep intervention groups will be asked additional questions during each questionnaire times so we can evaluate the interventions. These additional questions will take about ~10 minutes at each time.

**5. Can I choose my program?**

Participants will be randomly allocated to one of the two groups described above, which means that you will be unable to select which group you are allocated.

**6. I have been allocated to one of the sleep programs, can my partner or others involved in my baby's care also receive the intervention materials?**

Yes. We will discuss this with you after you are allocated to one of the groups, and work out an option that works best for you and your family. Your partner is also welcome to sign up to SHINE as a participant using this same consent form, and this does not affect how you and your family would receive the sleep program.

**7. Why are you not offering the sleep program to participants not having sleeping difficulties?**

Participants who do not currently experience sleeping difficulties can still participate in SHINE by completing the same questionnaires (see the section on All Participants above). Based on long-term data from a previous trial we conducted, individuals without sleeping problems at late pregnancy received very little benefits from sleep intervention. In contrast, those with sleeping difficulties at the start, had substantial number of benefits.

**8. My sleep is okay but my partner has sleeping difficulties. Can my partner receive the sleep program instead?**

Currently, the two sleep programs are designed to address sleeping difficulties specifically related to pregnancy and childbirth, with the support of the partner or another carer-giver. Therefore, we currently only enrol mothers and birthing parents with sleeping difficulties (along with their partners or other carers as support if applicable) in the sleep interventions. The SHINE project is trying to understand how fathers and non-birthing parents sleep during the perinatal periods, so we can design interventions specifically tailored for them in a future study. If your partner experience sleeping difficulties, we will provide information on how to seek treatment.

**9. What are the possible benefits?**

**For you as a participant.** Participants allocated to one of the two sleep intervention programs will receive information and strategies related to parental and infant sleep. Some of these strategies may have beneficial effects on your wellbeing, although there is no guarantee. There is no direct benefits to participants not receiving the sleep programs.

**For parents like you.** The information gathered from the study will be used to understand changes in sleep and the effectiveness of different programs for sleep health in new parents. Findings from this project will inform how perinatal sleep interventions could be further developed and implemented in perinatal care.

**10. What are the possible risks?**

There are no foreseen risks in participating in this study. It is possible that when completing some questionnaires about your feelings, you might think about things that upset you. In the event of such discomfort, please contact the research team on +61 3 9905 2464. If you prefer to speak to someone independent of this study about any distress you experience during your participation, we strongly encourage you to speak to your doctor, who will be able to link you to appropriate support. If you are in crisis, and would like to speak to a trained professional urgently, please call **Lifeline** on **13 11 14.**

**11. Do I have to take part in this research project?**

Participation in this study is voluntary. If you do not wish to take part, you are not obliged to. If you decide to take part and later change your mind, you are free to withdraw at a later stage.

If you do decide to withdraw, please complete the withdrawal form. To help ensure the results of the study can be measured properly, the researchers would like to keep your information that has been collected. If you do not want them to do this, please tell them.

If you are a patient at Monash Health or Royal Women's Hospital, your decision whether to take part or not, or to withdraw, WILL NOT affect your routine treatment, your relationship with those treating you, or your relationship with the Hospitals. Before making your decision, you can discuss any questions you have about the research project with a member of the research team.

**12. How will I be informed of the final results of this research project?**

If you wish, a summary of the study findings can be sent to you at the completion of the study. It is anticipated that this summary will be available within 18 months of study completion. In addition, at the end of the study, you may request more detailed findings by contacting us (see contact details).

**13. What will happen to information about me?**

Any information you provide us, including those via questionnaires and audio-recording, will remain confidential. To maintain your privacy, you will be assigned a numeric identification code. Any identifiable information you provide such as your name and contact information will be stored in a program that meets stringent criteria for data security of health-related information. Outside of this program, you will only be identified by your numeric code. All hard copies of data collected will be kept in secure locked filing cabinets at Monash University. All phone calls may be audio-recorded. Audio recordings will be stored securely with password protection. Trained researchers will extract coded information from these recordings, and store these codes securely with other information you have provided for analysis.

Only members of the SHINE research team will have access to information that might identify you (e.g., name, audio recording). Any publications or reports that arise from this study will include only combined results from many participants, so you or any information that might identify you (e.g., name, audio recording) will not be released.

The data collected will be retained for a minimum of 15 years. No more than seven years after the final publication, we will de-identify the data by removing names, date of birth, addresses/contact details, and any information that may link the data to you personally. These personally identifying data will be completely erased and destroyed. The de-identified database will be made publicly available through Monash University Bridges (or another research repository alike) to maximize the potential benefit to the scientific and research community.

If a member of our research team believes that there is a potential risk or danger posed to you or someone else, your contact details may be provided to support services. These may include, but not limited to your local specialist mental health service, or emergency services.

Should your partner be participating in SHINE for fathers and non-birthing parents, none of your information will be shared with them.

**14. Can I access research information kept about me?**

In accordance with relevant Australian and/or Victorian privacy and other relevant laws, you have the right to access the information collected and stored by the researchers about you. Please contact one of the researchers named at the end of this document if you would like to access your information.

**15. Is this research project approved?**

This project will be carried out according to the National Statement on Ethical Conduct in Human Research (2018) produced by the National Health and Medical Research Council of Australia. This statement has been developed to protect the interests of people who agree to participate in human research studies. Monash Health Ethics: RES-22-0000-379A

**16. Who can I contact?**

For further information concerning this project or if you have any problems that may be related to your involvement in the project, you can contact **+61 3 9905 2464,** email: **psych-** [**shine@monash.edu**](mailto:shine@monash.edu)

If you have any complaints about any aspect of the project, the way it is being conducted or any questions about being a research participant in general, then you may contact Monash Health Human Research Ethics Committee **+61 3 9594 4611**, email:

[**research@monashhealth.org**](mailto:research@monashhealth.org)
